# Supplementary material for: Toward characterizing cardiovascular fitness using machine learning based on unobtrusive data
Source: PLoS One. 2023 Mar 2;18(3):e0282398. doi: 10.1371/journal.pone.0282398 (PMC9980797; doi:10.1371/journal.pone.0282398)
Supplement: Attachment — Submitted filename: Response to Reviewers.docx [file pone.0282398.s003.docx]

Response to the Reviewer:

We appreciate all the comments from the reviewers. Improvements throughout the manuscript were made in accordance with the reviewers’ comments. The comments were extremely valuable for improving the quality of our manuscript. Please find below our responses and comments related to all the reviewers’ concerns.

Reviewer 1: Great work.

The use of digital technologies and Artificial intelligence is the growing world of knowledge and the making use of them through research adds another milestone to the advancement of the digital technologies in preventing future illness, which is by far more important in disease prevention.

Response: Thank you very much for your comment. We are so grateful to have such a positive comment.

Reviewer 2:

Line 49: the reference needs to be updated to reflect NCDs risk factors and not specifically CVD.

Response: Thank you for your comment. We agree with you, thus we added the following citation “Budreviciute A, Damiati S, Sabir DK, Onder K, Schuller-Goetzburg P, Plakys G, Katileviciute A, Khoja S and Kodzius R (2020) Management and Prevention Strategies for Non-communicable Diseases (NCDs) and Their Risk Factors. Front. Public Health 8:574111. doi: 10.3389/fpubh.2020.574111”.

Line 50 – 51: The authors seem to be including CVD as part of NCDs (paragraph 1) and now talking about NCDs impact on CVD. Please review.

Response: Thank you for your comment, we rewrote the statement following your comment.

Line 50: Not all NCDs are directly impacted by Cardiovascular health. The paper would benefit from clearly delineating CVD focus on not all NCDs.

Line 50: Suggest rewording cardiovascular health (CH) to cardiovascular fitness (CF). CH is very broad within which CF is focused area.

Response: Thank you for your comments. We corrected this sentence as you suggested. The term “cardiovascular healthy (CH)” was replaced by “Cardiovascular fitness (CF)”.

Line 54-56: The use of CPET in assessing cardiovascular health is one metrics for CVD risk. Only 1 of the three papers actually reference cardiorespiratory fitness. Also for subclinical CVD risk, CPET is not a scalable tool as there are other measures more closely aligned to overall risk.

Response: We would like to thank the reviewer for raising this issue. Although the term “Cardiovascular health and cardiovascular fitness” are used interchangeably sometimes we agree with the reviewer, so the manuscript was changed accordingly. Regarding the CPET application, we also added a sentence in line 64 to fit your suggestion.

Line 76: “pre-symptomatic detection of NCDs”. Please revise and focus on cardiovascular diseases.

Response: Thank you for your comment, according to your suggestion we added more information (in line 80) to the manuscript.

Sincerely,

The Authors
