# Supplementary material for: Toward characterizing cardiovascular fitness using machine learning based on unobtrusive data
Source: PLoS One. 2023 Mar 2;18(3):e0282398. doi: 10.1371/journal.pone.0282398 (PMC9980797; doi:10.1371/journal.pone.0282398)
Supplement: Attachment — Submitted filename: Response to Reviewers-30_01_23.docx [file pone.0282398.s004.docx]

Dear Editor,

We would like to thank you for allowing us to resubmit our manuscript. The modifications have been highlighted throughout the manuscript and their position in the manuscript has been reported in the response to referees. In addition, we also updated the values in table 1 after we double-checked our dataset which is now being shared in our submission. These new values did not change the statistical results as well as our study outcome.

We hope that now it is suitable for publication in PlosOne.

Response to the Reviewer:

We appreciate all the comments from the reviewers. Improvements throughout the manuscript were made in accordance with the reviewer comments. The comments were extremely valuable for improving the quality of our manuscript. Please find below our responses and comments related to all the reviewer concerns.

Reviewer 1: I have no specific comment to the authors.

All my comments and suggestions are well addressed and the authors did an incredible work.

Response: Thank you very much.

Reviewer 3: I read the manuscript interestingly and I found it so informative. Overall, the topic is interesting and important. Although this manuscript is well written and on a good subject, some issues need to be addressed:

Response: Thank you very much for this opportunity to improve our manuscript. We corrected all points that you mentioned.

Line 38: SVR stands for what? Please writhe the full form of abbreviation in the first appearance.

Response: Thank you for your comment, we rewrote the statement.

Line 37-40: Please re-write this section and provide separate results and conclusions.

Response: Thank you for your comment. We added the conclusion: “Therefore, we conclude that the cardiovascular fitness can be predicted by wearable technologies associated with machine learning during unsupervised activities of daily living. ”

Introduction is too long and boring.

Response: Thank you for your comment. We deleted some sentences in the introduction to make this section shorter and easier to read.

The significance level is 0.05 not 5%.

Response: Thank you for your comment, we corrected the statement.

Only 43 cases were included in the analysis. The sample size of 43 is too small to causal analysis and adjusting different variables. Explain it or provide a valid formula?

Response: We would like to thank the reviewer for raising this issue. Following your comment, we calculated the power of the sample a posteriori, considering as the main outcome the measured and predicted the $\dot{V}O_{2-max}$, as well as a correlation coefficient of 0.804 and a p value of 0.05. The test results are reported below (GPower 3.1 software):

**Exact -** Correlation: Bivariate normal model

**Options:**	exact distribution

**Analysis:**	Post hoc: Compute achieved power

**Input:**	Tail(s)	=	Two

Correlation ρ H1	=	0.804

α err prob	=	0.05

Total sample size	=	43

Correlation ρ H0	=	0

**Output:**	Lower critical r	=	-0.3007930

Upper critical r	=	0.3007930

Power (1-β err prob)	=	0.9999996

Thus, we found that for our sample size of 43 individuals, with an observed R of 0.804 ($\dot{V}O_{2-max}$and the $pred\dot{V}O_{2-max}$) and a p value of 0.05, the statistical power was 99.9%, which can be considered as a strong statistical power. As our SVR algorithm was successfully trained, we believe that its inputs carry information that explains the output from the model (knowledge extraction).

What was your criterion to select the variable to include in the models? The best recommended method is using directed acyclic graph (DAG) which helps you to find confounders, colliders and mediators.

Response: We thank the reviewer for this observation. We assume that the reviewer refers to the use of DAG for the hyperparameter selection process.

In this study, we used the SVR with an RFB Kernel, which means that we have a two-dimensional parameter space. Due to the low complexity of searching hyperparameters in this parameter space, we adopted a simple, effective, and well-known method named Grid Search [1]. In short, given a set of values for the variables that comprise the model, i.e., the parameters C and Gamma, the Grid Search algorithm makes a direct search on a set of all trials, which is formed by assembling every possible combination of values for the C and Gamma. We agree with the reviewer that this approach does not guarantee the best values for the parameters [1], and thus better approaches for doing that, as pointed out by the reviewer, can potentially give us better results, we would like to keep this hyperparameter search simple due mainly because of the number of samples that we have available for training. Recall that, in several applications, the use of Grid Search often leads to the choice of hyperparameter values that are effective in practice. Therefore, it is one of the widely used approaches for hyperparameter definition in machine learning tasks.

In order to attend to the comment of the reviewer, we added a paragraph in the manuscript to inform the reader about the mentioned limitation and the suggested methodology for future improvement of this work.

[1] Bergstra, James, and Yoshua Bengio. "Random search for hyper-parameter optimization." Journal of machine learning research 13.2 (2012).

Please provide a list of algorithms used in the SVM.

Response: Thank you for your comment. Please follow the list of algorithms used in the SVM.

-	R.-E. Fan, K.-W. Chang, C.-J. Hsieh, X.-R. Wang, and C.-J. Lin. LIBLINEAR: A library for large linear classification Journal of Machine Learning Research 9(2008), 1871-1874.

-	C.-C. Chang and C.-J. Lin. LIBSVM: a library for support vector machines. ACM Transactions on Intelligent Systems and Technology, 2:27:1--27:27, 2011.

Sincerely,

The Authors
